# Effectiveness of seasonal influenza vaccines in children – a systematic review and meta-analysis

**DOI:** 10.3325/cmj.2013.54.135

**Published:** 2013-04

**Authors:** Ivana Lukšić, Sarah Clay, Rachel Falconer, Dražen Pulanić, Igor Rudan, Harry Campbell, Harish Nair

**Affiliations:** 1 Institute of Public Health “Dr Andrija Štampar,” Zagreb, Croatia; 2 Centre for Population Health Sciences, University of Edinburgh, Medical School, Edinburgh, Scotland, UK; 3 Clinical Hospital Centre Zagreb, Zagreb, Croatia; 4 Faculty of Medicine Osijek, J.J. Strossmayer University of Osijek, Osijek, Croatia; 5 Public Health Foundation of India, New Delhi, India; * These authors contributed equally.

## Abstract

**Aim:**

To assess the efficacy and effectiveness of seasonal influenza vaccines in healthy children up to the age of 18 years.

**Methods:**

MedLine, EMBASE, CENTRAL, CINAHL, WHOLIS, LILACS, and Global Health were searched for randomized controlled trials and cohort and case-control studies investigating the efficacy or effectiveness of influenza vaccines in healthy children up to the age of 18 years. The studies were assessed for their quality and data on the outcomes of influenza-like illness, laboratory-confirmed influenza, and hospitalizations were extracted. Seven meta-analyses were performed for different vaccines and different study outcomes.

**Results:**

Vaccine efficacy for live vaccines, using random effects model, was as follows: (i) for similar antigen, using per-protocol analysis: 83.4% (78.3%-88.8%); (ii) for similar antigen, using intention to treat analysis: 82.5 (76.7%-88.6%); (iii) for any antigen, using per protocol analysis: 76.4% (68.7%-85.0%); (iv) for any antigen, using intention to treat analysis: 76.7% (68.8%-85.6%). Vaccine efficacy for inactivated vaccines, for similar antigen, using random effects model, was 67.3% (58.2%-77.9%). Vaccine effectiveness against influenza-like illness for live vaccines, using random effects model, was 31.4% (24.8%-39.6%) and using fixed-effect model 44.3% (42.6%-45.9%). Vaccine effectiveness against influenza-like illness for inactivated vaccines, using random effects model, was 32.5% (20.0%-52.9%) and using fixed-effect model 42.6% (38.3%-47.5%).

**Conclusions:**

Influenza vaccines showed high efficacy in children, particularly live vaccines. Effectiveness was lower and the data on hospitalizations were very limited.

Influenza virus causes an acute respiratory tract infection, which results in considerable morbidity and mortality worldwide (1,2). Influenza A genus is classified into many different serotypes; H1N1 and H3N2 notably cause disease in humans. Population groups at particular risk of influenza include children, pregnant women, the elderly, and patients with co-morbidities (3). Prevention of influenza is a major health issue worldwide, but providing protection by vaccination is challenging because each season the disease is caused by different strains of the virus. Current recommendations for influenza vaccination include people over the age of 65, pregnant women, people with chronic respiratory illness, and those in long-stay residential care homes (4). Influenza vaccines are not typically recommended for use in healthy children. Vaccination of healthy children aged from 6 to 24 months has been advocated in the USA, since 2004, which has gradually included children up to 59 months, and the current recommendation is to vaccinate all children from 6 months up to 19 years – with particular emphasis on children under the age of 5 or with chronic illnesses (5). No influenza vaccines have been approved for use in children younger than 6 months (6).

There are two types of vaccine available – live and inactivated, both of which can be used in children. They contain three different virus strains: two influenza A (H1N1-like and H3N2-like) and one influenza B, which have to be administered annually. Live vaccines are administered via an intranasal spray, whereas inactivated vaccines are injected, either intramuscularly or intradermally. Inactivated influenza vaccines contain killed virus, which makes them safer for use in pregnant women and those with underlying medical conditions; they cannot induce mild signs and symptoms of influenza like live vaccines. Vaccines can be adjuvanted to improve their immunogenicity (7). Inactivated influenza vaccines have been successfully adjuvanted and their safety and immunogenicity proven for use in children (8). It has also been hypothesized that vaccinating children against influenza has indirect benefits, such as reducing influenza-related morbidity and mortality in the elderly population (9). This study aimed to assess which vaccines were most appropriate for use in healthy children from 6 months to 18 years of age, by critically and systematically reviewing the evidence on their efficacy and effectiveness and performing meta-analysis of the available information.

## Methods

### Search of the literature

We aimed to identify all randomized control trials (RCTs) and cohort or case-control studies investigating the effectiveness or efficacy of influenza vaccines in reducing the incidence of influenza-like illness (ILI) or laboratory-confirmed influenza in children. In order to achieve this we searched Ovid MEDLINE (In-Process & Other Non-Indexed Citations and Ovid MEDLINE 1946 to Present), Embase + Embase Classic (1947 to present), the Cochrane Central Register of Controlled Trials (CENTRAL), Cumulative Index of Nursing and Allied Health Literature (CINAHL), World Health Organisation Library Information System (WHOLIS), Latin American and Caribbean Health Sciences Literature (LILACS), and Global Health Library (1910 to December 2011) using different combinations including the following search terms: influenza vaccines, attenuated vaccines, live vaccines, vaccination, human influenza, child, preschool-child, infant, influenza-like illness, hospitalization.

**Figure 1:**
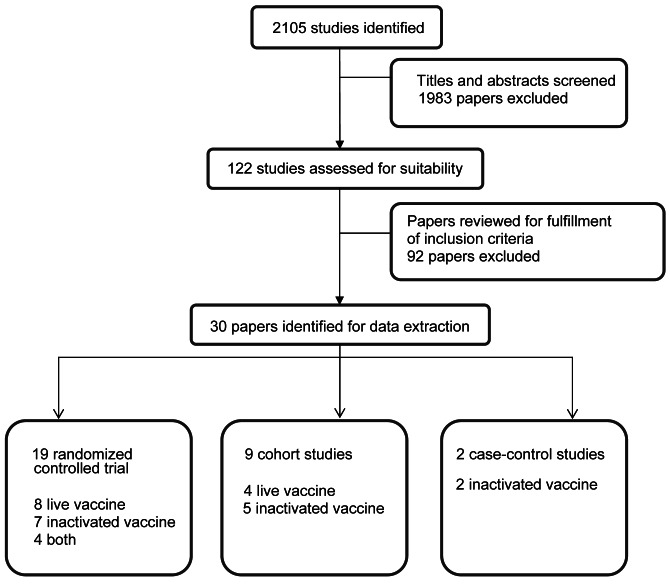
A summary of the literature review.

After elimination of duplicates, results of searches from all databases combined identified 2105 potentially relevant articles. The titles and abstracts were screened and studies measuring only the safety or immunogenicity of vaccines were excluded, and so were the studies on pandemic influenza vaccines, studies assessing efficacy or effectiveness of vaccines in adults and children with asthma, and review articles. This process left 122 articles that required a more detailed evaluation, 92 of which did not fulfill the inclusion criteria. The number of studies that met all of the inclusion criteria was 30 (Figure 1).

### Selection criteria

This review included RCTs, cohort studies, and case-control studies carried out in any country until the end of December 2011. Studies eligible for inclusion were those assessing any preparation of seasonal influenza vaccine, administered via any route to healthy participants up to and including the age of 18 years. For inclusion, studies had to have a comparison to a control group that received either an alternative vaccine, placebo, or no intervention. Outcome measures considered for inclusion were cases of influenza or ILI confirmed either clinically or by laboratory techniques, hospitalizations due to influenza, and preventive efficacy or effectiveness of vaccines. For the purposes of this review, efficacy was defined as the ability of the vaccine to reduce the number of laboratory-confirmed cases of illness caused by influenza infection. Effectiveness was defined as the ability of the vaccine to reduce clinical cases of symptomatic influenza-like illnesses.

We excluded the studies that assessed vaccines in children with chronic illnesses, such as asthma. Searches also produced a considerable number of studies that did not record clinical outcomes, but rather defined the presence of infection using serological data, such as antibody titers. Those studies were also excluded from the assessment of effectiveness.

### Data analysis

We extracted information on the main characteristics of the studies on live vaccines (Table 1) and inactivated vaccines (Table 2) for all 30 studies. In order to judge the quality of the studies, further information about randomization, allocation concealment, intention to treat analysis, and blinding was extracted. We assessed the quality of studies using standard GRADE criteria (Supplementary Table S1(supplementary material)) and extracted detailed definitions on the outcomes used in the studies (Supplementary Table S2(supplementary material)). Potential confounding factors and any resulting adjustments were also recorded. In the studies that reported overall vaccine efficacy or effectiveness (VE), calculating it as:

**Table 1:** Characteristics of studies on live vaccines

Author (year of publi-cation)	Location	Age of study population	Study design	Type of vaccine
Alexandrova (1986)	USSR	3-15 years	RCT	Live cold-adapted recombinant bivalent vaccine of influenza A 47/25/1 (H1N1) and 47/7/2 (H3N2).
Belshe (1998)	USA	15-71 months	RCT	Live attenuated cold-adapted trivalent vaccine containing influenza A/Texas/36/91-like (H1N1), A/Wuhan/359/95-like (H3N2) and B/Harbin/7/94-like viruses.
Belshe (2000)	USA	26-85 months	RCT	Live attenuated cold-adapted trivalent vaccine containing influenza A/Shenzhen/227/95-like (H1N1), A/Wuhan/359/95 (H3N2) and B/Harbin/94-like viruses.
Beutner (1979)	USA	7-14 years	RCT	Live recombinant influenza vaccine (X-42)
Clover (1991)	USA	3-18 years	RCT	Live attenuated bivalent cold recombinant vaccine containing A/Bethesda/1/85 (H3N2) and A/Texas/1/85 (H1N1).
Khan (1996)	Russia	9-12 years	RCT	Live, trivalent attenuated, cold-adapted vaccine containing A/Leningrad/92/89 (H1N1), A/Zakarpatje/354/89 (H3N2) and B/Yamagata/16/88
Longini (2000)	USA	15-71 months	RCT	Live attenuated, cold-adapted influenza vaccine. The vaccine attenuated strains were matched to the antigens as recommended for the trivalent inactivated influenza vaccines by the Food and Drug Admini-stration for the 1996-97 and 1997-98 influenza seasons
Neto (2009)	South Africa, Brazil, Argentina	6 to <36 months	RCT	Live attenuated influenza vaccine, containing reassortant influenza virus strains containing the haemagglutinin and neuraminidase antigens of influenza virus strains recommended by the WHO
Rudenko (1993)	Russia, Novgorod	7-14 years	RCT	Live attenuated vaccine containing, in 1989: A/Taiwan/1/86 (H1N1)-like, A/Sichuan/2/87 (H3N2)-like and in 1990: A/Taiwan/1/86 (H1N1)-like, A/Shanghai/11/87 (H3N2) and B/Victoria/2/87-like.
Rudenko (1996)	Russia, Cuba	3-15 years	RCT	Live, cold-adapted influenza vaccine. (No further details provided).
Tam (2007)	South East Asia	12 to <36 months	RCT	Live CAIV-T, in year 1: A/New Caledonia/20/99 (H1N1), A/Sydney/05/97 (H3N2) and B/Yamanashi/ 166/98 and in year 2: A/New Caledonia/20/99, A/Panama/2007/99 and B/Yamanashi/166/98. Composition was planned to be antigenically representative of the WHO recommendations for the Northern hemisphere each year.
Vesikari (2006)	Europe	6 to <36 months	RCT	Live CAIV-T containing in year 1: A/New Caledonia/20/99 (H1N1), A/Sydney/05/97 (H3N2) and B/Yamanashi/166/98 and in year 2: A/New Caledonia/ 20/99, A/Panama/2007/99 and B/Victoria/504/2000.
Gaglani (2004)	USA	1.5-18 years	Cohort study	Live, attenuated, cold-adapted influenza vaccine containing A/Beijing/262/95 (H1N1) [A/New Caledonia/20/99 (H1N1) in 2000], A/Sydney/05/97 (H3N2) and B/Beijing/184/93-like strains.
Halloran (2003)	USA	1.5-18 years	Cohort study	Live-attenuated, trivalent, cold-adapted vaccine containing strains A/Beijing/262/95 (H1N1) [A/New Caledonia/20/99 (H1N1) in 2001], A/Sydney/5/97 (H3N2) and B/Beijing/184/93-like.
Piedra (2005)	USA	1.5-18 years	Cohort study	Live trivalent attenuated vaccine (CAIV-T) containing, in 1998-99: A/Beijing/262/95 (H1N1) [A/Caledonia/20/99 in 2000-01], A/Sydney/05/97 (H3N2) and B/Beijing/184/93-like.
Piedra (2007)	USA	5-18 years	Cohort study	Live trivalent attenuated influenza vaccine containing A/New Caledonia/20/99 (H1N1)-like, A/Panama/2007/99 (H3N2)-like, B/Hong Kong/330/2001-like

**Table 2:** Characteristics of studies on inactivated vaccines

Author (year of publication)	Location	Age of study population	Study design	Type of vaccine
Beutner (1979)	USA	7-14 years	RCT	Inactivated Port Chalmers (H3ChN2Ch) influenza vaccine (X-41).
Clover (1991)	USA	3-18 years	RCT	Inactivated trivalent vaccine containing A/Chile/83 (H1N1), A/Mississippi/85 (H3N2) and B/Ann Arbor/86
Colombo (2001)	Italy	1-6 years	RCT	Inactivated trivalent subvirion vaccine containing A/Johannesburg/33/94-like, A/Singapore/6/86-like and B/Beijing/184/93-like.
Cowling (2010)	Hong Kong, Kowloon	6-15 years	RCT	Inactivated TIV seasonal vaccine containing strains A/Brisbane/59/2007 (H1N1)-like, A/Brisbane/10/2007(H3N2)-like and B/Florida/4/2006.
Gruber (1990)	USA	3-18 years	RCT	Inactivated TIV containing A/Chile/83 (H1N1), A/Philippines/82 (H3N2) and B/USSR/83 strains.
Hoberman (2003)	USA	6-24 months	RCT	Inactivated trivalent subvirion influenza vaccine in 1999-200, containing: A/Beijing/262/95 (H1N1), A/Sydney/15/97 (H3N2) and B/Yamanashi/166/98 and in 2000-01: A/New Caledonia/20/99 (H1N1), A/Panama/2007/99 (H3N2) and B/Yamanashi/166/98
Khan (1996)	Russia	9-12 years	RCT	Inactivated, trivalent split-virus influenza vaccine containing A/Taiwan/1/86 (H1N1), A/Shanghai/16/89 (H3N2) and B/Yamagata/16/88
Maeda (2004)	Japan	6-24 months	RCT	Inactivated influenza vaccine containing, in 1999-2000: A/Beijing/262/95 H1N1), A/Sydney/5/97 (H3N2) and B/Shandong/7/97. In 2000-01: A/New Caledonia/20/99, A/Panama/2007/99 and B/Johannesburg/5/99. In 2001-02: A/New Caledonia/20/99, A/Panama/2007/99 and B/Johannesburg/5/99
Principi (2003)	Italy	0.5-5 years	RCT	Inactivated virosomal influenza vaccine (no information on strains included)
Rudenko (1993)	Russia	7-14 years	RCT	Inactivated (strains contained are the same as in the live vaccine, detailed above).
Vesikari (2011)	Germany, Finland	6 to <72 months	RCT	Inactivated, TIV containing, in 2007-08: A/Solomon Islands/3/2006 (H1N1), A/Wisconsin/67/2005 (H3N2) and B/Malaysia/2506/2004 and in 2008-09: A/Brisbane/59/2007, A/Brisbane/10/2007 and B/Florida/4/2006 and TIV MF59 emulsion adjuvant
Fujieda (2008)	Japan	Under 6 years	Cohort study	Inactivated TIV containing A/New Caledonia/20/99 (H1N1), A/Panama/2007/99(H3N2) and B/Shandong/7/97.
Heikkinen (1991)	Finland	1-3 years	Cohort study	Inactivated, trivalent subvirion influenza vaccine containing A/Taiwan/1/86 (H1N1), A/Sichuan/2/87 (H3N2) and B/Victoria/2/87.
Katayose (2011)	Japan	0.5-5 years	Cohort study	Inactivated TIV - vaccine components were decided yearly, by National Institute of Infectious Diseases, Japan based on WHO recommendations
Salleras (2006)	Spain	3-14 years	Cohort study	Inactivated virosomal subunit influenza vaccine (no further details)
Yamaguchi (2010)	Japan	6-12 years	Cohort study	Inactivated trivalent influenza vaccine containing A/New Caledonia/20/99 (H1N1), A/Hiroshima/52/2005 (H3N2) and B/Malaysia/2506/2004
Joshi (2009)	USA	6-59 months	Case-control	Inactivated trivalent influenza vaccine strains contained over the 8-year study period varied according to ACIP recommendations
Kelly (2011)	Australia, Western Australia	0.5-5 years	Case-control	Inactivated trivalent influenza vaccine containing A/Solomon Islands/3/2006 (H1N1)-like strain, an A/Brisbane/10/2007 (H3N2)-like strain and B/Florida/4/2006-like strain

VE = 100% - odds ratio (OR); or VE = 100% - risk ratio (RR),

with 95% confidence intervals (CIs) provided, results were entered directly into the data extraction table. Wherever studies had reported OR or RR, the overall VE and CIs were calculated. In the studies that only reported the number of ILIs (or laboratory-confirmed influenza cases) in vaccinated individuals and controls, the OR was calculated and overall VE and CIs recorded. Studies were then classified by outcomes, to allow separate assessment of efficacy and effectiveness. Tables were created to compare the efficacy or effectiveness of live and inactivated vaccines (Table 3-6). From each table, the data were used to perform 7 separate meta-analyses: for efficacy of live vaccines (based on similar antigen and per protocol analysis, similar antigen and intention to treat analysis, any antigen and per protocol analysis, and any antigen and intention to treat analysis), efficacy of inactivated vaccines (based on similar antigen, any analysis), effectiveness of live vaccines, and effectiveness of inactivated vaccines (supplementary Figures S1-S7(supplementary material)). Two results were reported for each meta-analysis, based on random effects model and fixed effects model, and presenting pooled meta-estimates with 95% confidence intervals. For all computations, Stata 11.2 (StataCorp, TX, USA) was used.

**Table 3:** A review of efficacy estimates for live vaccines (PPT– per protocol; ITT – intention to treat).

Author (year of publication)	Study design	Type of vaccine	Control	Age of study population	Overall vaccine efficacy (VE)	Lower confidence interval limit (for overall VE)	Upper confidence interval limit (for overall VE)
Belshe (1998)	RCT	Live	Placebo	15-71 months	0.93	0.88	0.96
Belshe (2000)	RCT	Live	Placebo	26-85 months	0.87	0.78	0.93
Beutner (1979)	RCT	Live	Placebo	7-14 years	0.62	0.44	0.87
Clover (1991)	RCT	Live	Placebo	3-18 years	0.65	0.31	1.36
Longini (2000)	RCT	Live	Placebo	15-71 months	1996-97: 0.90 1997-98: 0.85	0.51 0.47	1.59 1.53
Neto (2009)	RCT	Live	Placebo	6 to <36 months	Year 1, similar antigen: 0.74 Year 1, any antigen: 0.72 Year 2, similar antigen: 0.74 Year 2, any antigen: 0.47	0.64 0.62 0.33 0.15	0.81 0.80 0.91 0.67
Tam (2007)	RCT	Live	Placebo	12 to <36 months	Year 1, PPT, similar antigen: 0.73 Year 1, PPT, any antigen: 0.70 Year 1, ITT, similar antigen: 0.70 Year 1, ITT, any antigen: 0.68 Year 2, PPT, similar antigen: 0.84 Year 2, PPT, any antigen: 0.64	0.63 0.61 0.60 0.59 0.70 0.44	0.81 0.77 0.78 0.75 0.92 0.77
Vesikari (2006)	RCT	Live	Placebo	6 to <36 months	Year 1, PPT, similar antigen: 0.85 Year 1, PPT, any antigen: 0.86 Year 1, ITT, similar antigen: 0.84 Year 1, ITT, any antigen: 0.84 Year 2, PPT, similar antigen: 0.89 Year 2, PPT, any antigen: 0.86 Year 2, ITT, similar antigen: 0.89 Year 2, ITT, any antigen: 0.85	0.74 0.76 0.73 0.74 0.82 0.79 0.83 0.78	0.92 0.92 0.91 0.90 0.93 0.91 0.93 0.90
Halloran (2003)	Cohort study	Live	No intervention	1.5-18 years	Combined, A (H1N1) and B: 0.91	-0.34	0.99

**Table 6:** A review of effectiveness estimates for inactivated vaccines (ILI – influenza-like illness; ARI – acute respiratory infection; URTI – upper respiratory tract infection; LRTI – lower respiratory tract infection)

Author (year of publication)	Study design	Type of vaccine	Control	Age of study population	Overall vaccine effectiveness	Lower confidence interval limit	Upper confidence interval limit
Colombo (2001)	RCT	Inactivated	No intervention	1-6 years	0.77	0.45	1.31
Cowling (2010)	RCT	Inactivated	Placebo	6-15 years	ILI: 0.08 ARI: 0.01	0.04 0.01	0.16 0.02
Gruber (1990)	RCT	Inactivated	Placebo	3-18 years	0.85	0.41	1.74
Principi (2003)	RCT	Inactivated	No intervention	0.5-5 years	URTIs: 0.33 LRTI: 0.22 ILI: 0.26	0.19 0.08 0.18	0.57 0.34 0.36
Rudenko (1993)	RCT	Inactivated	Placebo	7-14 years	Year 1: 0.33 Year 2: 0.27	0.15 0.20	0.38 0.34
Fujieda (2008)	Cohort study	Inactivated	No intervention	<6 years	0.24	0.12	0.34
Heikkinen (1991)	Cohort study	Inactivated	No intervention	1-3 years	0.18	0.13	0.25
Salleras (2006)	Cohort study	Inactivated	No intervention	3-14 years	ILI: 0.75	0.61	0.84

## Results

The retained 30 studies comprised 19 RCTs (10-28), 9 cohort studies (29-37), and 2 case-control studies (38,39). There were 9 studies that assessed the efficacy of live vaccines, 8 of which were RCTs (11-14,20,22,26,27) and 1 was cohort study (31). Eight studies evaluated the effectiveness of live vaccines: 4 RCTs (10,19,24,25) and 4 cohort studies (30,31,34,35). Thirteen studies assessed the efficacy of inactivated vaccines: 7 RCTs (13,14,16-18,21,28), 4 cohort studies (32,33,36,37), and 2 case-control studies (38,39). Eight studies assessed the effectiveness of inactivated vaccines: 5 RCTs (15-17,23,24) and 3 cohort studies (29,32,36). The results were expected to show higher efficacy (which was assessed against proven influenza infection) than effectiveness (which would be measured against any cause of respiratory illness).

### Assessment of quality of the evidence

To assess the quality of studies we used the GRADE criteria (40) (supplementary material(supplementary material)). In terms of study design, 19 RCTs were used along with 11 observational studies (9 cohort and 2 case-control). Observational studies, in general, have lower validity than RCTs, and overall study quality was moderate. Results for the efficacy of inactivated vaccines were not particularly consistent, which may partly be due to strain matching of vaccines to viruses. Directness of the studies was good, with age groups, interventions used, and definition of outcomes all showing the expected directness when compared to the final outcomes. The heterogeneity of ILI definitions weakened directness slightly.

### Efficacy of live and inactivated vaccines

Vaccine efficacy for live vaccines, using random effects model, was as follows: (i) for similar antigen, using per-protocol analysis: 83.4% (78.3%-88.8%); (ii) for similar antigen, using intention to treat analysis: 82.5 (76.7%-88.6%); (iii) for any antigen, using per protocol analysis: 76.4% (68.7%-85.0%); (iv) for any antigen, using intention to treat analysis: 76.7% (68.8%-85.6%). Vaccine efficacy for inactivated vaccines, for similar antigen, using random effects model, was 67.3% (58.2%-77.9%). The results of fixed-effects model consistently showed slightly greater efficacy than the results of random-effects model (Supplementary Figure S1-S5(supplementary material)).

One study (33) recorded the efficacy of inactivated vaccines in children up to 6 years old only, and presented the results by age group. The efficacy was consistently high in the 6 months to 1 year age group. Only one study (28) assessed the efficacy of an adjuvanted inactivated vaccine and compared it to a non-adjuvanted inactivated vaccine. The adjuvanted vaccine showed a greater efficacy than control and also non-adjuvanted vaccine. The efficacy of the adjuvanted vaccine in this study was 80%, which is comparable to the efficacy of live vaccines. The effect of inactivated vaccines on reducing hospitalization was only recorded in one study (33), which showed the overall efficacy of 71% in reducing hospitalizations from influenza A infection in children aged 6 months to 6 years and of 72% for influenza B infection.

### Effectiveness of live and inactivated vaccines

Vaccine effectiveness against influenza-like illness for live vaccines, using random effects model, was 31.4% (24.8%-39.6%) and using fixed-effect model 44.3% (42.6%-45.9%). Vaccine effectiveness against influenza-like illness for inactivated vaccines, using random effects model, was 32.5% (20.0%-52.9%) and 42.6% (38.3%-47.5%) using fixed-effect model (Supplementary Figure S6-S7(supplementary material)). Clearly, the lower values for effectiveness than for efficacy reflect the differences in outcomes used in the studies. The estimates of efficacy were based on studying the outcomes which influenza vaccines were designed to protect against, while for assessing effectiveness the outcomes were less specific.

## Discussion

This review showed a high efficacy of influenza vaccines against influenza infection, but a considerably lower effectiveness. There was a slight difference in favor of live vaccines in efficacy, but hardly any in effectiveness. Two studies demonstrated the efficacy of live vaccines in children under 2 years of age (22,27), which was an important contribution to available information on this age group. Expectedly, both live and inactivated vaccines were more efficacious against infection with strains of influenza virus that were antigenically similar to strains contained in the vaccine (22,26-28), highlighting the need for good strain-matching for the vaccines that are being delivered in large programs. Adjuvanted inactivated vaccines showed efficacy almost as good as that of live vaccines, although this result came from only one study (28). These conclusions are in line with previously conducted systematic reviews on influenza vaccines (41), suggesting that there is a growing agreement about the key technical indicators, and that the future research challenges will be more focused on achieving more equitable impact of the available preventive interventions (42).

This report has several limitations. We performed meta-analyses of the studies that were matched for the type of vaccine (live or inactivated), antigen used (similar or any), and analysis used (per protocol or intention to treat). Still, studies that were grouped together varied in study designs, case definitions, and age ranges applied (Supplementary material(supplementary material)). Moreover, we excluded several papers in Russian (43-49), because it was not possible for us to assess full text articles and we already felt that we had enough evidence. For a more in depth review, we could also consider wider aspects of influenza vaccination, such as the cost-effectiveness and safety of implementing vaccination programs for different age groups, the effect that vaccination may have on reducing absence from school and parents’ absence from work, and acceptability of vaccine to providers and end-users. There have been studies researching the indirect beneficial effect that vaccinating children has on morbidity and mortality from influenza in the elderly (9), which could be another interesting research area.

In many studies, there was a lack of information on the composition of the vaccine used and its matching to the circulating strains of influenza viruses for that season. This is vital as it inevitably will have a significant effect on the efficacy of vaccines. Influenza vaccines will not have effect against antigenically dissimilar viruses. There are also some limitations imposed by the nature of research available in the area of influenza vaccines. There were no studies that directly compared live and inactivated vaccines, although such studies would allow an analysis of the efficacy and effectiveness of the vaccines in a comparable context. There was a considerable heterogeneity in the outcome definitions used in the studies, particularly with reference to ILI definitions. Studies also differed according to methods of collecting data on ILIs; in some studies cases were diagnosed by general practitioners or pediatricians on visits to a clinic and in others according to parental reports of symptoms.

The main strength of this review, compared to other relevant literature, is that it summarized the available information on this issue and provided an evidence base from which further research could be conducted. It exposed differences in the quality and methodological approaches to studies, such as the definition of outcomes and study designs and gaps in information provided for certain age groups. It strengthened the existing knowledge, providing a summary of the data on the effect of live and inactivated vaccines in preventing influenza in children, in whom it showed comparable results to healthy adults. Areas for future research have also been highlighted, such as the direct comparison of the efficacy of live and inactivated vaccines, more systematic research into benefits of each type of vaccine for different age groups, and the need for a wider evidence base supporting potentially promising adjuvanted inactivated influenza vaccines (50).

## 

**Table 4:** A review of efficacy estimates for inactivated vaccines.

Author (year of publication)	Study design	Type of vaccine	Control	Age of study population	Overall vaccine efficacy (VE)	Lower confidence interval limit (for overall VE)	Upper confidence interval limit (for overall VE)
Beutner (1979)	RCT	Inactivated	Placebo	7-14 years	0.82	0.55	1.23
Clover (1991)	RCT	Inactivated	Placebo	3-18 years	0.74	0.29	1.88
Cowling (2010)	RCT	Inactivated	Placebo	6-15 years	0.56	0.25	1.23
Gruber (1990)	RCT	Inactivated	Placebo	3-18 years	Against influenza B: 0.75	0.34	1.64
Hoberman (2003)	RCT	Inactivated	Placebo	6-24 months	0.66	0.34	0.82
Maeda (2004)	RCT	Inactivated	No intervention	6-24 months	Against influenza A: 0.45	0.18	1.10
Vesikari (2011)	RCT	Inactivated	Placebo	6 to <72 months	Year 1, any antigen: 0.43 Year 1, similar antigen: 0.45 Year 2, any antigen: 0.40 Year 2, similar antigen: 0.41	0.15 0.16 -0.06 -0.89	0.61 0.64 0.66 0.58
Heikkinen (1991)	Cohort study	Inactivated	No intervention	1-3 years	Against influenza A: 0.85	0.32	2.24
Katayose (2011)	Cohort study	Inactivated	No intervention	0.5-5 years	Against influenza A: 0.53	0.41	0.63
Salleras (2006)	Cohort study	Inactivated	No intervention	3-14 years	Against influenza A: 0.88	0.49	0.97
Yamaguchi (2010)	Cohort study	Inactivated	No intervention	6-12 years	0.82	0.60	1.12
Joshi (2009)	Case-control	Inactivated	Negative laboratory test for influenza-like illness (ILI)	6-59 months	0.86	0.29	0.97
Kelly (2011)	Case-control	Inactivated	Negative laboratory test for ILI	0.5-5 years	0.58	0.09	0.81

**Table 5:** A review of effectiveness estimates for live vaccines

Author (year of publication)	Study design	Type of vaccine	Control	Age of study population	Overall vaccine effectiveness	Lower confidence interval limit	Upper confidence interval limit
Alexandrova (1986)	RCT	Live	Placebo	3-15 years	0.55	0.51	0.60
Khan (1996)	RCT	Live	Placebo	9-12 years	0.47	0.35	0.61
Rudenko (1993)	RCT	Live	Placebo	7-14 years	Year 1: 0.48 Year 2: 0.41	0.22 0.14	0.58 0.54
Rudenko (1996)	RCT	Live	No intervention	3-15 years	Year 1: 0.36 Year 2: 0.48	0.33 0.45	0.39 0.50
Gaglani (2004)	Cohort study	Live	No intervention	1.5-18 years	Year 1: 0.22 Year 2: 0.21	0.11 0.10	0.32 0.31
Halloran (2003)	Cohort study	Live	No intervention	1.5-18 years	0.18	0.11	0.24
Piedra (2005)	Cohort study	Live	Placebo	1.5-5 years	0.07	0.05	0.09
Piedra (2007)	Cohort study	Live	No intervention	5-18 years	0.42	0.35	0.50
