# Supplementary material for: Effectiveness of seasonal influenza vaccines in children – a systematic review and meta-analysis
Source: Croat Med J. 2013 Apr;54(2):135–45. doi: 10.3325/cmj.2013.54.135 (PMC3662362; doi:10.3325/cmj.2013.54.135)

Supplementary online material:

Table S1. Assessment of study quality using GRADE criteria.

**Author (year of publication)**	**Study design - quality of evidence**	**Study Quality**	**Consistency**	**Directness** (for outcome definitions, see table 2)
Alexandrova (1986)	RCT - high	Good - allocation concealed, assessment blinded but not intention to treat (ITT) analysis	Moderate consistency	Excellent - appropriate age group, intervention and outcomes for effectiveness
Belshe (1998)	RCT - high	Good - allocation concealed, assessment blinded but not ITT analysis	Good consistency	Excellent - appropriate age group, intervention and outcomes for efficacy
Belshe (2000)	RCT - high	Good - allocation concealed, assessment blinded but not ITT analysis	Moderate consistency	Excellent - appropriate age group, intervention and outcomes for efficacy
Beutner (1979)	RCT - high	Excellent - allocation concealed, assessment blinded and ITT analysis	Good consistency	Excellent - appropriate age group, intervention and outcomes for efficacy and compares both live and inactivated vaccines
Clover (1991)	RCT - high	Moderate - unclear whether allocation concealed, asssessment blinded but not ITT analysis	Good consistency	Good - appropriate age group, intervention and outcomes for efficacy but only against influenza A infection
Colombo (2001)	RCT - high	Moderate - no allocation concealment, unclear whether assessment blinded, ITT analysis	Moderate consistency	Excellent - appropriate age group, intervention and outcomes for effectiveness, clear definition of ILI
Cowling (2010)	RCT - high	Excellent - allocation concealed, assessment blinded and ITT analysis	Good for efficacy, moderate for effectiveness	Good - appropriate age group, intervention and outcomes for efficacy and effectiveness
Gruber (1990)	RCT - high	Good - allocation concealed, assessment blinded, unclear wheter ITT analysis	Good for efficacy, poor for effectiveness	Good - appropriate age group, intervention and outcomes for efficacy but only against influenza B infection and effectiveness but no definition of ILI
Hoberman (2003)	RCT - high	Good - no allocation concealment, assessment blinded and ITT analysis	Moderate consistency	Excellent - appropriate age group, intervention and outcomes for efficacy
Khan (1996)	RCT - high	Poor - unclear whether allocation concealed and assessment blinded, no ITT analysis	Good consistency	Moderate - appropriate but limited age group, appropriate intervention and outcomes for effectiveness but based on school absence
Longini (2000)	RCT - high	Good - allocation concealed, assessment blinded but no ITT	Good consistency	Moderate - appropriate age group and intervention, outcome

**Author (year of publication)**	**Study design - quality of evidence**	**Study Quality**	**Consistency**	**Directness** (for outcome definitions, see table 2)
		analysis		vague, no detail on detection of cases/surveillance of subjects
Maeda (2004)	RCT - high	Moderate - unclear whether allocation concealed, assessment blinded but no ITT analysis	Moderate consistency	Moderate - appropriate age group and intervention but outcome of efficacy only against influenza A infection
Neto (2009)	RCT - high	Good - allocation concealed and assessment blinded but no ITT analysis	Good consistency	Excellent - appropriate age group, intervention, good outcome definition and looks at antigenically similar and any strain infections.
Principi (2003)	RCT - high	Moderate - no allocation concealment, unclear whether assessment blinded, ITT analysis	Good consistency	Excellent - appropriate age group, intervention, outcome definition very suitable for measuring effectiveness
Rudenko (1993)	RCT - high	Moderate - unclear whether allocation concealed and assessment blinded, no ITT analysis	Good consistency	Good - appropriate age group, intervention and outcome for effectiveness but surveillance/detection of cases only by way of school absence
Rudenko (1996)	RCT - high	Moderate - unclear whether allocation concealed, assessment was blinded but unclear whether ITT	Good consistency	Good - appropriate age group, intervention and effectiveness outcome but uncclear whether only detected at school, no ILI definition
Tam (2007)	RCT - high	Excellent - allocation concealment, assessment blinded and ITT analysis	Good consistency	Excellent - appropriate age group, intervention and good, clear outcome definition for efficacy
Vesikari (2006)	RCT - high	Moderate - no allocation concealment, unclear whether assessment blinded, ITT analysis	Good consistency	Excellent - appropriate age group, intervention and very clearly defined methods of case detection
Vesikari (2011)	RCT - high	Moderate - unclear whether allocation concealed and assessment blinded, no ITT analysis	Good consistency	Good - appropriate age group, intervention, good outcome for efficacy but no definition of 'illness' leading to lab diagnosis, looks at an adjuvanted vaccine
Fujieda (2008)	Cohort study - low	Moderate - unclear whether allocation concealedand assessment blinded, no ITT analysis	Good consistency	Moderate - appropriate age group, intervention and outcome for effectiveness but no clear ILI definition
Gaglani (2004)	Cohort study - low	Poor - no allocation concelament, assessment not blinded and no ITT analysis	Good consistency	Excellent - appropriate age group, broad, appropriate intervention and clear outcome

**Author (year of publication)**	**Study design - quality of evidence**	**Study Quality**	**Consistency**	**Directness** (for outcome definitions, see table 2)
				definition for effectiveness
Halloran (2003)	Cohort study - low	Moderate - no allocation concealment, unclear whether assessment blinded, unclear whether ITT analysis	Good consistency	Excellent - wide, appropriate age group, appropriate intervention and clear definitions of efficacy and effectiveness-both measured
Heikkinen (1991)	Cohort study - low	Moderate - no allocation concealed, assessment blinded, no ITT analysis	Good consistency	Excellent - appropriate, if narrow age group, appropriate intervention, clear outcome definitions for both efficacy and effectiveness
Katayose (2011)	Cohort study - low	Poor - unclear whether allocation concealed, assessment not blinded and no ITT analysis	Good consistency	Good - appropriate age group, intervention and outcomes for efficacy although only measured for influenza A infection
Piedra (2005)	Cohort study - low	Poor - no allocation concealed, unclear whether assessment blinded, no ITT analysis	Moderate consistency	Excellent - appropriate, wide age group, intervention and good outcome definitions
Piedra (2007)	Cohort study - low	Poor - no allocation concealed, unclear whether assessment blinded, no ITT analysis	Good consistency	Good - appropriate age group and intervention, clear definitions of outcome measures
Salleras (2006)	Cohort study - low	Moderate - unclear whether allocation concealed or assessment blinded, no ITT analysis	Doos for efficacy, moderate for effectiveness	Excellent - appropriate age group and intervention, very well defined outcome measures
Yamaguchi (2010)	Cohort study - low	Poor - no allocation concealment, unclear whether assessment blinded, no ITT analysis	Good consistency	Excellent - appropriate age group and intervention, clear outcome definitions and separately classified illness caused by influenza A and B
Joshi (2009)	Case-control - low	Moderate - unclear whether allocation concealed, assessment was blinded, no ITT analysis	Good consistency	Good - appropriate age group and intervention, good outcome definitions
Kelly (2011)	Case-control - low	Moderate - no allocation concealment, assessment blinded, no ITT analysis	Good consistency	Excellent - appropriate age group, intervention and very clear case definitions

Table S2. Outcome definitions of included studies.

Author (year of publication)	Outcome definition
Alexandrova (1986)	Incidence of influenza and acute respiratory disease during influenza epidemic. Comparison of the influenza morbidity rates among vaccine and control groups of children were based on clinical diagnosis during the epidemic period.
Belshe (1998)	Influenza defined as any illness detected by active surveillance associated with positive culture for wild type influenza virus 28 days after the first dose and any time after the second dose during the influenza A H3N2 and B epidemic. After outbreak of influenza in the community parents were contacted and reminded to notify if the subject had symptoms suspected to be caused by influenza : fever, runny nose, nasal congestion, sore throat, cough, headache, muscle aches, chills, vomiting, suspected or confirmed otitis media, decreased activity, irritability, wheezing, shortness of breath, and pulmonary congestion. Attempted to collect viral culture specimens within four days after the onset of any illness.
Belshe (2000)	Primary end-point of efficacy - first episode of culture-confirmed influenza occurring in an individual child after revaccination. Subtype specific efficacy (A and B). Influenza - any illness detected by active surveillance associated with positive culture for wild-type influenza virus. Incidences of flu-like illness detected by surveillance - diagnoses included LRT disease (physician-diagnosed croup, bronchitis, pneumonia or wheezing) and otitis media with or without concomitant fever.
Beutner (1979)	The extent and nature of clinical illness in the vaccinees during the subsequent outbreaks of natural influenza infection were evaluated by examination of all sick children within 24 hours. Influenza confirmed by isolation of influenza virus strains from serum.
Clover (1991)	Influenza A infection: Febrile illnesses (with temperature >38°C) : including upper or lower respiratory tract illness, otitis media, influenza-like illness. Afebrile illnesses: When community surveillance indicated that influenza virus was spreading in the community (influenza A/Taiwan/86), weekly telephone contacts to families were made to evaluate respiratory illnesses. Home or clinic visits were scheduled for physical examination and collection of nasal washes or swab specimens for viral isolation. An illness was attributed to influenza A infection if influenza virus was isolated during the illness. Illnesses were characterized by review of records which included date of onset, symptoms, physical signs diagnosis of each contact.
Colombo (2001)	Influenza-like illness: fever (rectal temperature >38.5°C) and cough or sore throat lasting at least 72 hours.
Cowling (2010)	Serologically confirmed infection indicated by a 4-fold or greater increase in antibody titer.
	ILI - temperature ≥37.8°C plus cough or sore throat
	ARI - self-reported symptoms; at least any 2 of fever ≥37.8°C, chills, headache, sore throat, cough, phlegm, nasal congestion, runny nose and muscle or joint pain
Fujieda (2008)	ILI: acute febrile illness occurring during the highest epidemic period in each study area (ILI, not influenza as claimed by the authors). Fever reported as below 38, between 38 and 39 and 39 or more (no description of how temp was taken)
Gaglani (2004)	Medically attended acute respiratory illness: upper and lower respiratory tract illnesses, otitis media and sinusitis - clinic and emergency department visits included. Health care providers then obtained a throat swab or a nasal wash for an influenza virus culture
Gruber (1990)	Influenza-like illness caused by influenza B virus infection, confirmed by viral isolation from nasal washes or throat swabs taken from subjects experiencing any respiratory symptoms (no further detail)
Halloran (2003)	MAARI - any URI, LRI, sinusitis, otitis media. Influenza A (H1N1) and B confirmed by throat swab or nasal wash from any child presenting with a history of fever and any respiratory illness
Heikkinen (1991)	Children symptomatic of a respiratory tract infection or fever had nasopharyngeal mucus specimen for rapid viral antigen detection of influenza A virus infection

Author (year of publication)	Outcome definition
Hoberman (2003)	Throat cultures positive for any influenza virus in children presenting with signs and symptoms of a URTI and fever (at least 38°C) or AOM or both
Joshi (2009)	Laboratory-confirmed influenza virus infection and medically attended influenza illness outpatient/ER visit or inpatient hospitalisation
Katayose (2011)	Influenza-positive rapid diagnostic test in patients with acute respiratory infectious symptoms and fever >38°C during surveillance period
Kelly (2011)	Children presenting to GP or emergency department with ILI and testing positive for influenza virus. ILI defined as a documented fever with oral (or aural) temperature ≥38°C (or axillary temperature >37.5°C) with at least one acute respiratory symptom or sign.
Khan (1996)	First school absence with physician's diagnosis of acute respiratory disease or influenza. (Influenza=acute respiratory illness within influenza season (clinical diagnosis)
Longini (2000)	Culture-confirmed influenza (lacking definition of surveillance of cases)
Maeda (2004)	Children presenting to hospital with a febrile illness (>38.0°C) had throat swabs taken and immunoassay membrane test done to detect influenza A virus antigen
Neto (2009)	Primary - First episode of culture-confirmed influenza illness caused by community-acquired subtypes antigenically similar to those contained in the vaccine
	Secondary - first episode of culture-confirmed influenza caused by any community-acquired subtypes
Piedra (2005)	MAARI visits to clinics, emergency rooms and hospitals, including those for otitis media and sinusitis, upper respiratory tract illness and lower respiratory tract illness
Piedra (2007)	MAARI - otitis media and sinusitis, URT illness, LRT illness, presenting to clinics, emergency departments and hospitals
Principi (2003)	Children presenting to emergency departments or primary care paediatricians with symptoms of respiratory tract infection. Influenza was then identified by culture and/or polymerase chain reaction
Rudenko (1993)	Acute respiratory disease' or 'influenza' as recorded on medical certificates for absence from school
Rudenko (1996)	Influenza and acute respiratory diseases diagnosed by a nurse in each school or kindergarten
Salleras (2006)	Acute febrile respiratory illness - detected by questionnaire filled out by parents.
	Influenza-like illness - paediatrician-attended cases of fever >38.5°C for at least 72 hours, cough or sore throat.
	Influenza A cases - children with ILI (as defined above) who had pharyngeal/nasal samples positive for influenza A on RT-PCR
Tam (2007)	Culture-confirmed influenza illness from viral isolation of nasal swabs taken from subjects presenting with ILI
Vesikari (2006)	Culture-confirmed influenza illness from nasal swabs taken from children exhibiting fever (rectal temperature of ≥38°C or axillary temperature of ≥37.5°C), wheezing, shortness of breath, pulmonary congestion, pneumonia or ear infection (suspected diagnosis of AOM). Nasal swab also required if subjects showed 2 or more of the following: runny nose or nasal congestion, sore throat, cough, muscle aches, chills, headache, irritability, decreased activity or vomiting.
Vesikari (2011)	Influenza confirmed with strain identification by PCR of nasopharyngeal aspirates or swabs obtained from children who became ill during the study period (no definition of 'illness' provided)
Yamaguchi (2010)	Case of influenza confirmed by a positive rapid diagnostic test then classified as influenza A or B. No details of detecting cases for rapid diagnostic testing.

Figure S1. Vaccine efficacy for live vaccines, similar antigen, using per protocol analysis (using data from Table 3): D+L indicates meta-estimates from random effects model; I+V indicates meta-estimates from fixed effects model; Halloran (2003) was excluded from meta-analysis, as this is a cohort study (but with Halloran (2003) study included, meta-estimate is 83.4 (78.5, 88.8)).

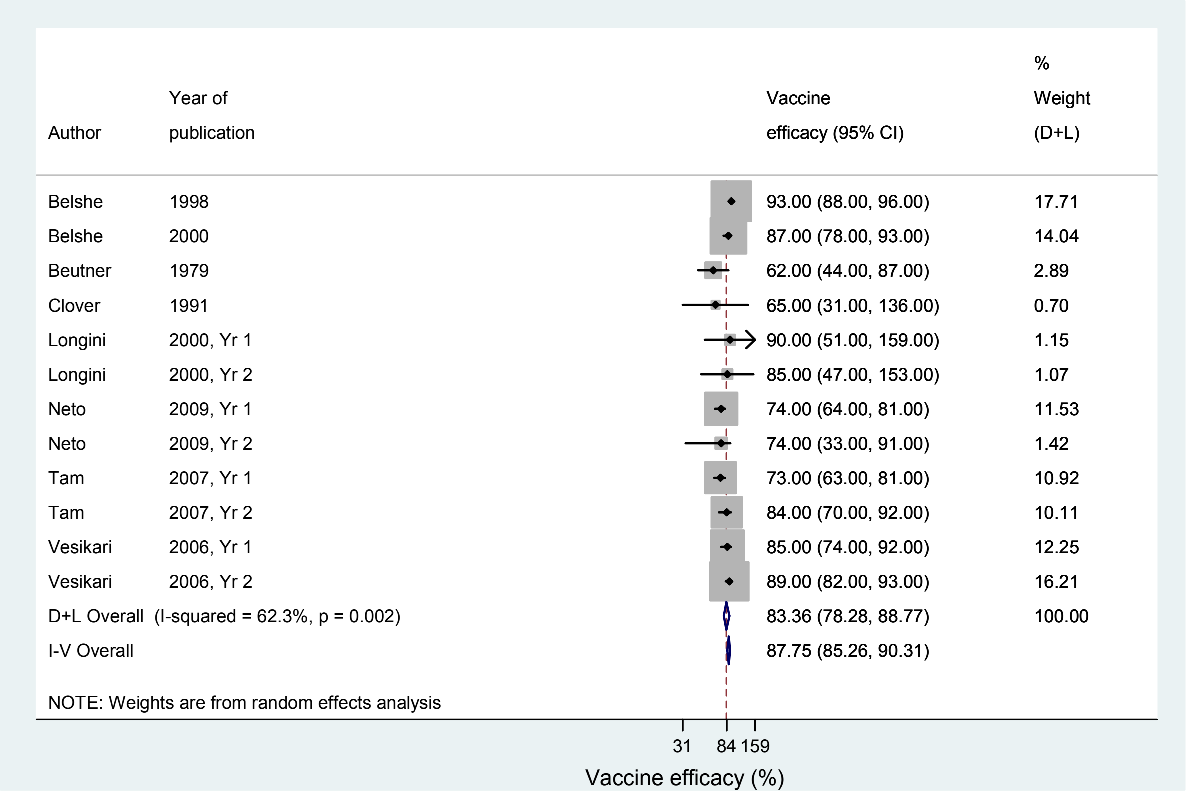

Figure S2. Vaccine efficacy for live vaccines, similar antigen, using intention to treat analysis (using data from Table 3): D+L indicates meta-estimates from random effects model; I+V indicates meta-estimates from fixed effects model.

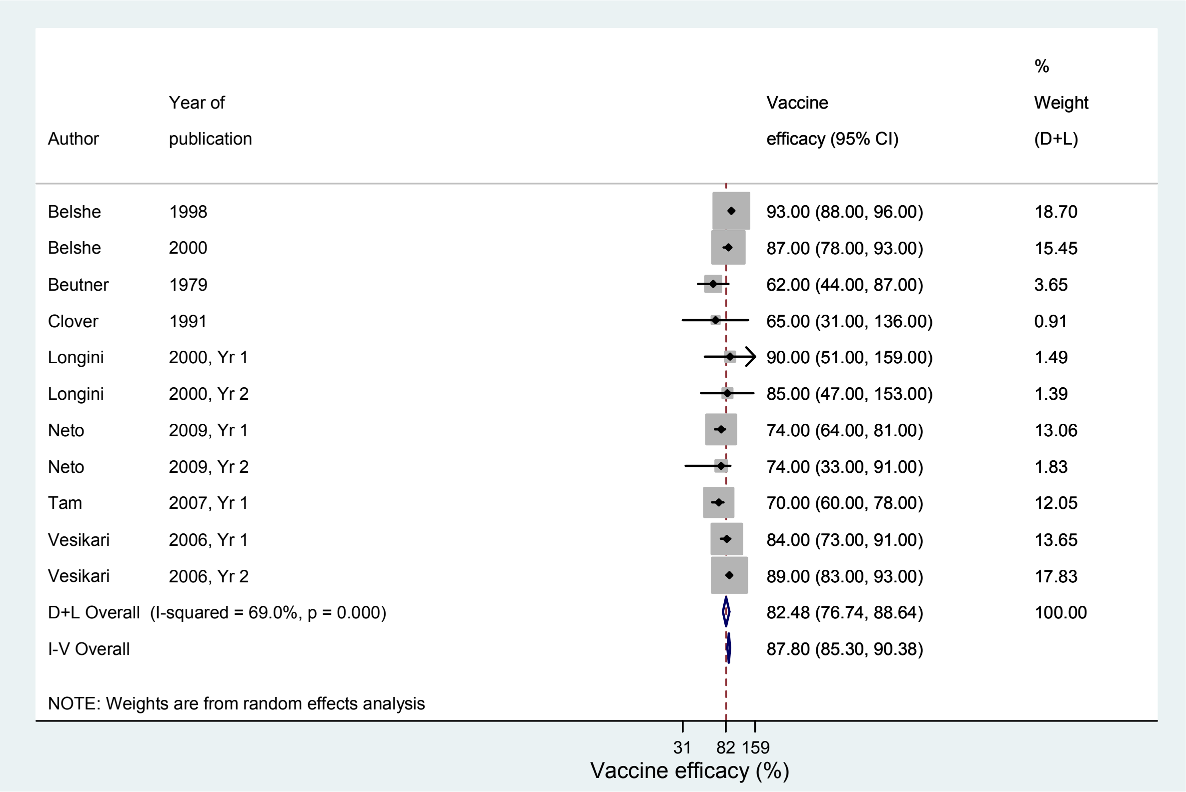

Figure S3. Vaccine efficacy for live vaccines, any antigen, using per protocol analysis (using data from Table 3): D+L indicates meta-estimates from random effects model; I+V indicates meta-estimates from fixed effects model.

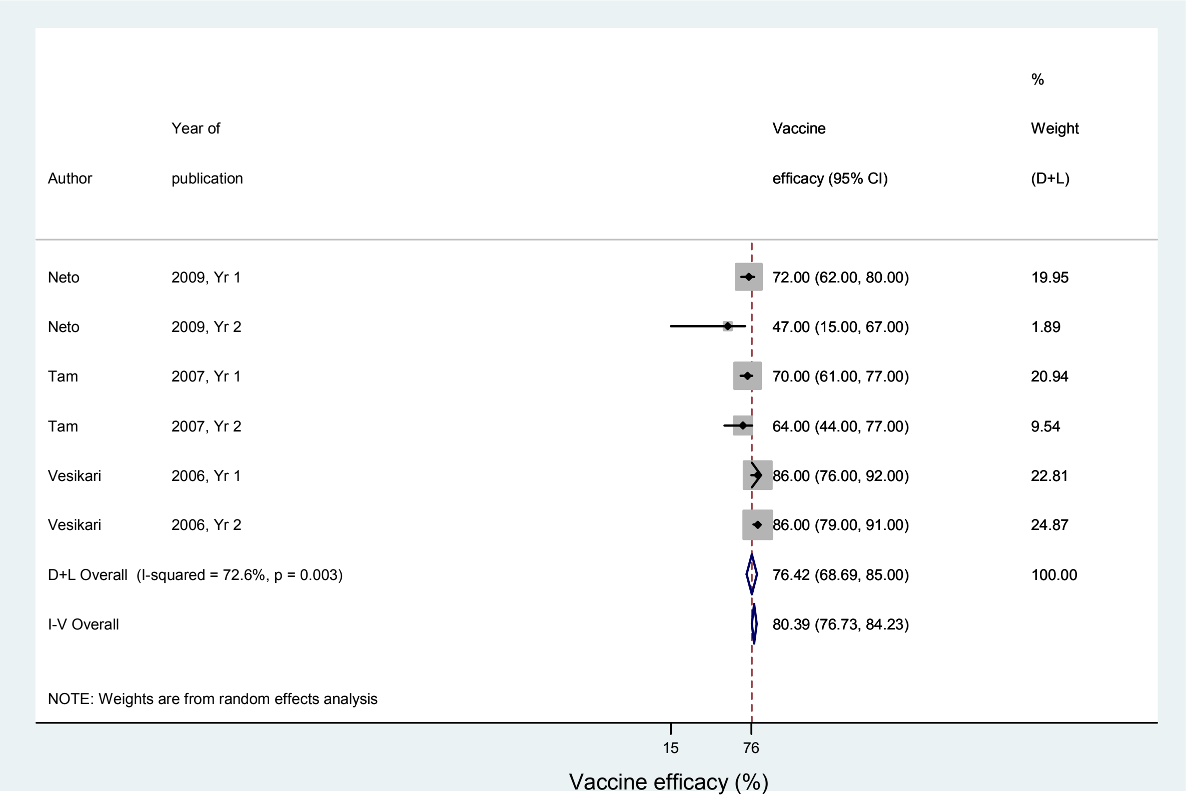

Figure S4. Vaccine efficacy for live vaccines, any antigen, using intention to treat analysis (using data from Table 3): D+L indicates meta-estimates from random effects model; I+V indicates meta-estimates from fixed effects model.

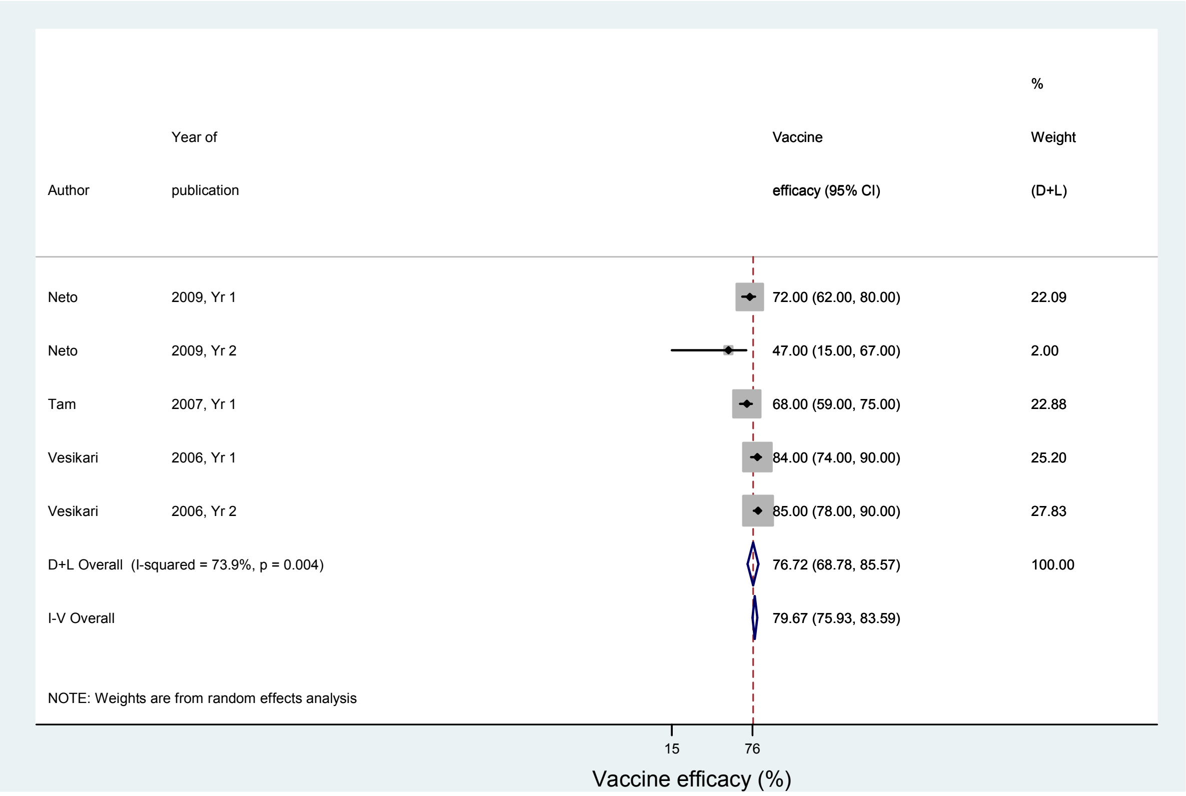

Figure S5. Vaccine efficacy (VE) for inactivated vaccines, similar antigen (using data from Table 4): D+L indicates meta-estimates from random effects model; I+V indicates meta-estimates from fixed effects model. Only 1 study (Vesikari) reported VE for any antigens. When VE for any antigen from this study was used in this analysis (i.e. VE for any antigen instead of similar antigen) then VE for RCT from random effects was 65.88 (52.43, 82.80), and overall VE was 67.32 (57.98, 78.16).

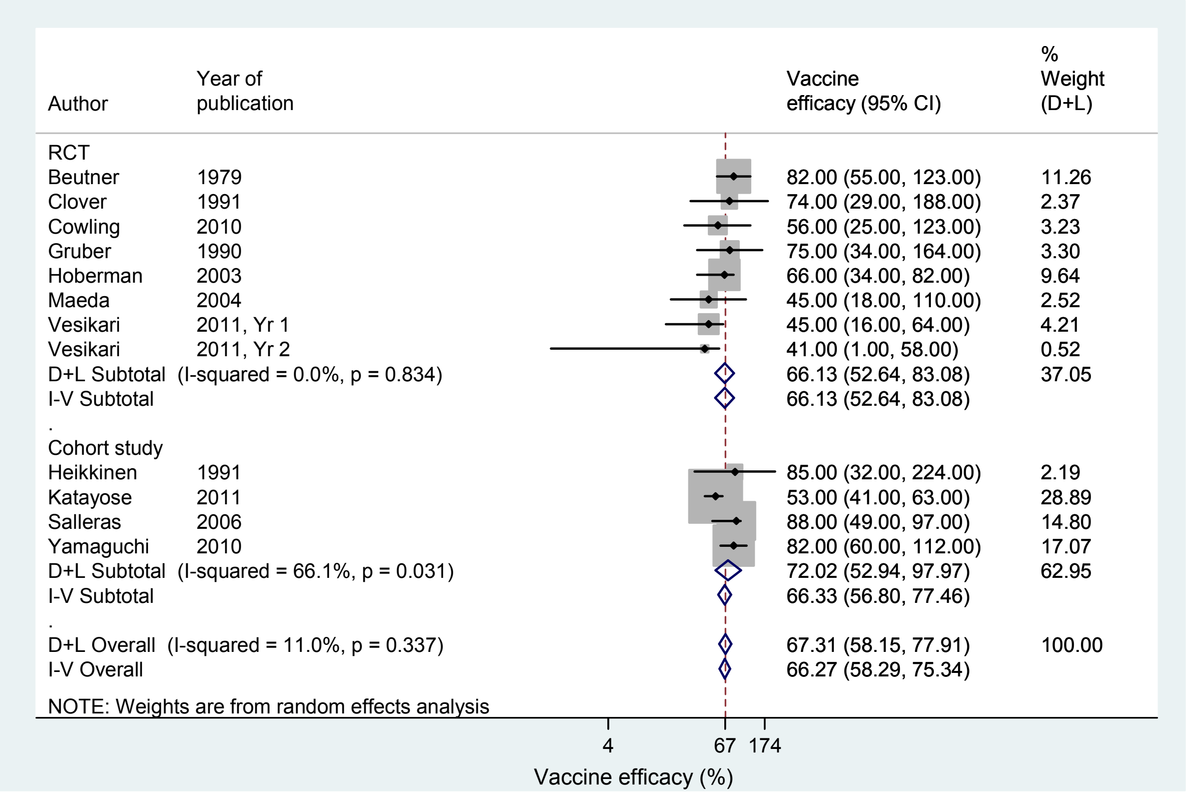

Figure S6. Vaccine effectiveness for live vaccines (using data from Table 5): D+L indicates meta-estimates from random effects model; I+V indicates meta-estimates from fixed effects model.

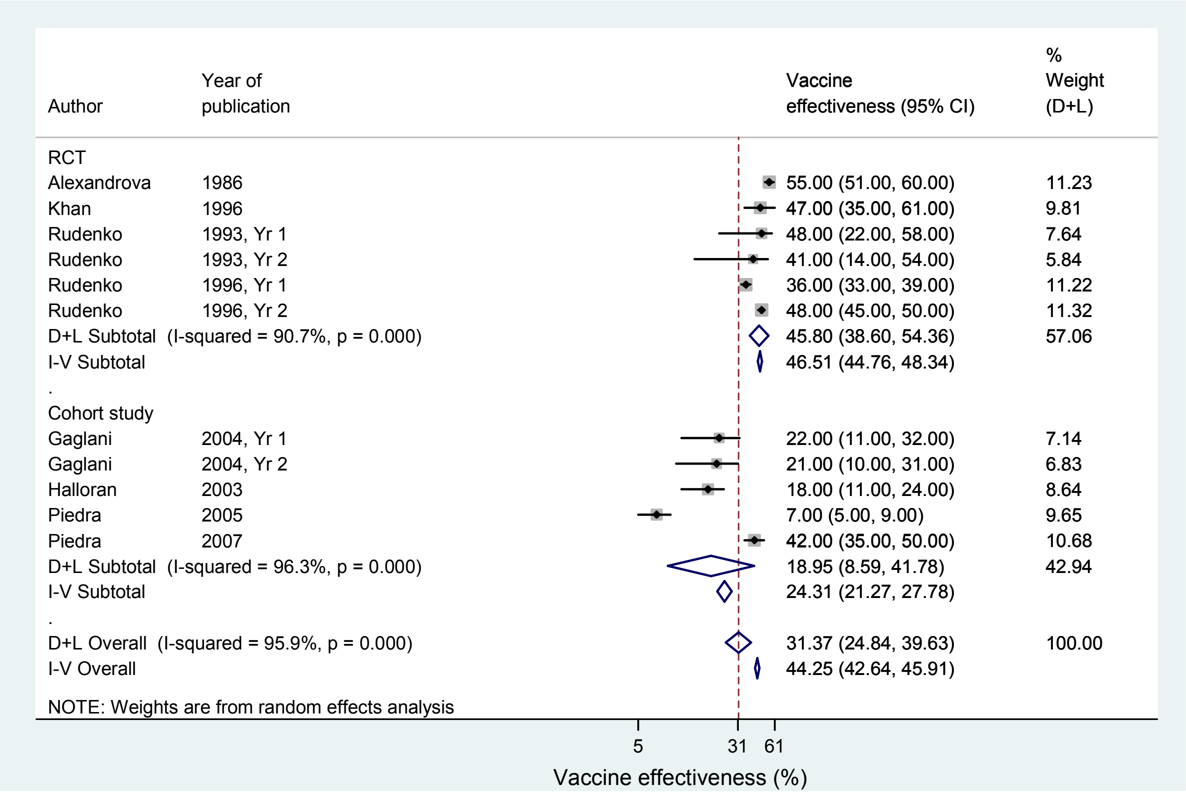

Figure S7. Vaccine effectiveness for inactivated vaccines for influenza-like illness (using data from Table 6): D+L indicates meta-estimates from random effects model; I+V indicates meta-estimates from fixed effects model. Only those studies that were reporting influenza like illness, or respiratory disease and influenza without segregating for acute respiratory infection (ARI) / acute lower respiratory infection (ALRI) / upper respiratory tract infection (URTI) have been included in the meta-analysis assuming all these studies are reporting ILI.

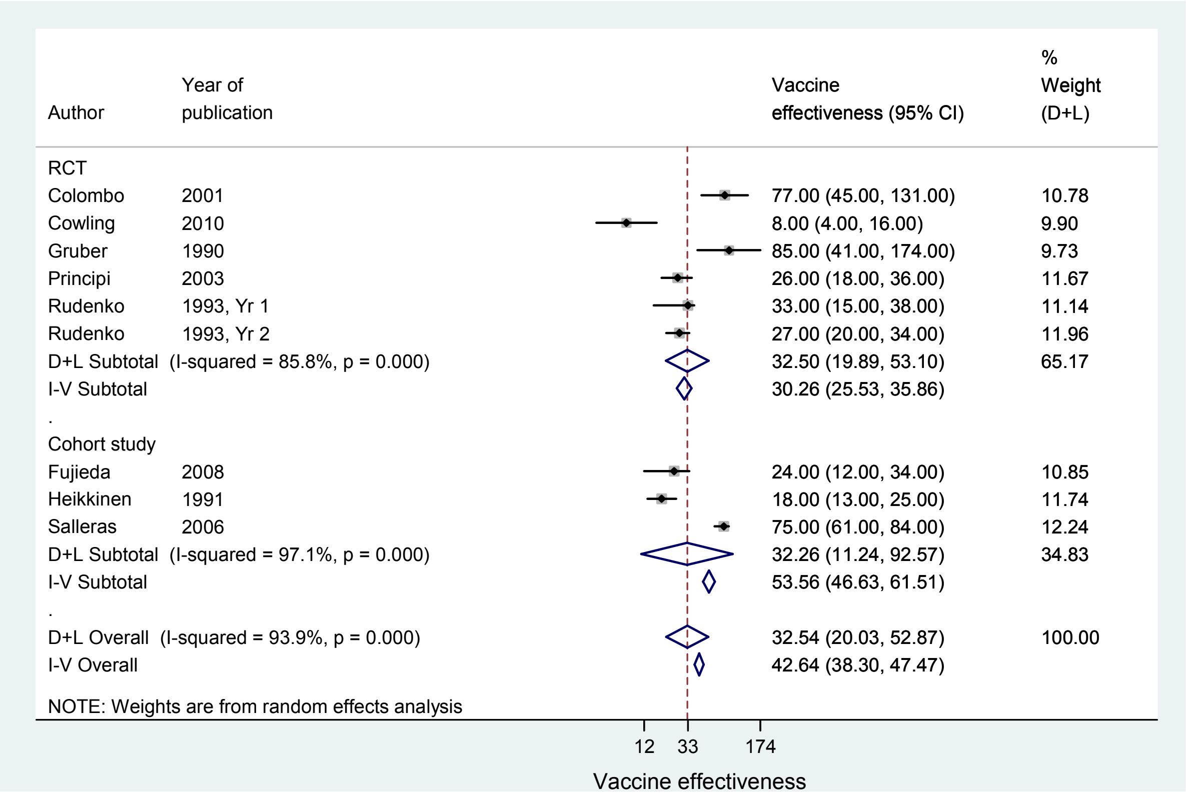

Supplement: Supplementary material [file CroatMedJ_54_s009.pdf]
